# Prevalence of *Shigella* among diarrheic children under-5 years of age attending at Mekelle health center, north Ethiopia

**DOI:** 10.1186/s13104-015-1752-1

**Published:** 2015-12-15

**Authors:** Atsebaha Gebrekidan Kahsay, Zelalem Teklemariam

**Affiliations:** Department of Microbiology and Immunology, Institute of Biomedical Sciences, College of Health Sciences, Mekelle University, P.O. Box: 1871, Mekelle, Tigray Ethiopia; Department of Medical Laboratory Sciences, College of Health and Medical Sciences, Haramaya University, P.O.Box. 331, Harar, Ethiopia

**Keywords:** *Shigella*, Children under-5 years of age, Diarrhea, Mekelle health center

## Abstract

**Background:**

Shigellosis is recognized as a major global public health problem especially in developing countries particularly in children under-5 years of age. Therefore; the objective of this study was to determine the prevalence of *Shigella* among diarrheic children under-5 years of age attending at Mekelle health center, north Ethiopia.

**Methods:**

A cross-sectional study was conducted among diarrheic children under-5 years of age from March to May, 2012. Structured questionnaire was used to collect the data. Study participants were recruited by convenience sampling technique. *Shigella* was isolated and identified using standard bacteriological techniques.

**Results:**

A total of 241 study participants were included in the study. The overall prevalence of *Shigella* in this study was 13.3 % (32/241). High prevalence of *Shigella* (22.6 %) was revealed from the age group of 12–23 months. No *Shigella* was isolated from the age group of 0–5 months. Majority of the isolates of *Shigella* were from bloody and mucoid diarrhea.

**Conclusion:**

There was high prevalence of *Shigella* infection in this study. Children among the age group of 12–23 months were highly affected. Therefore; responsible bodies should work hard on preventive measures to reduce or eradicate the problem occurred due to shigellosis.

## Background

Shigellosis is one of the main public health problems throughout the world. More than 99 % of the world annual episodes of *Shigella* (with 1.1 million deaths) were reported from developing countries. About 69 % of the world annual episodes and 61 % of all the deaths were shown in children under-5 years of age [1]. High incidence rates of *Shigella* infection were also revealed among children under-5 years of age in United States of America [2], Europe [3] and six Asian countries (China, Thailand, Vietnam, Pakistan, Bangladesh and Indonesia) [4]. The occurrence of Shigellosis in Canada was shown in increasing order from 1.8/100,000 in 2009 to 2.82/100,000 in 2012 [5].

*Shigella* was reported by the Multisite birth cohort study (MAL-ED) as one of the most causative agent of the second life of children in developing countries [6]. It was also described by the Global Enteric Multi-central Study (GEMS) as one of the four contributing agents (rotavirus, *Cryptosporidium*, enterotoxigenic *Escherichia coli—*ST-ETEC) of moderate to severe diarrhea among children under-5 years in Sub Saharan Africa [7].

The prevalence of *Shigella* in children under-5 years of age was variable from country to country. Half of the positive isolates of *Shigella* in one study in China [8] and about 79 % in Eastern Nepal [9] were shown among children under-5 years of age. The positivity rate of *Shigella* in Brazil [10] among 6–24 months and in China [11] among 3–4 years was 73 % and 32/1000/year respectively. Nearly half of the positive isolates of *Shigella* revealed from Eritrea were among children under-5 years of age [12]. The proportion of *Shigella* in Mucus, bloody and watery diarrhea was 53, 30 and 17 % respectively as indicated in the study in Central Republic of Africa [13].

The prevalence of *Shigella* in Ethiopia: Hawassa, Gondar, and Jimma was 7 [14], 4.57 [15] and 2.3 % [16] respectively. The positivity rate of *Shigella,* carried out in Gondar, among children under-5 years of age was 31.1 % [17].

An estimation of 133,000 annual episodes of diarrhea was reported from 2 months to 5 years children in Mekelle city. Above 38 % of the episodes reported from children in Mekelle city contained blood in their stool [18]. There was not published study on the magnitude of *Shigella* which causes diarrhea in Mekelle city. Therefore this study tried to determine the prevalence of *Shigella* among children under-5 years of age attending at Mekelle health center, north Ethiopia.

## Methods

### Study area and period

Mekelle is the capital city of Tigray regional state which is located 784 km north of Addis Ababa. It is with a total population of 261,000 [19]. This study was conducted among diarrheic children under-5 years of age attending at Mekelle health center from March to May, 2012.

### Exclusion criteria

Children under-5 years of age who were critically ill or under antibiotic taking for diarrhea during the period of sample collection were excluded from the study.

### Sample size determination

Sample size was calculated using single proportion formula by taking (P = 0.195) [20], 5 % margin of error, and Zα/2 = 1.96 for 95 % level of confidence.

$${\text{n}}= \frac{({{{{\text{Z}}\upalpha /2})^2 \times {\text{P}}\, ( { 1 -{\text{ P}}} )}}}{{{\text{d}}^{ 2} }} = 2 4 1$$

### Sampling technique

Consecutive study participants having diarrhea were recruited using convenience sampling technique.

### Data collection and laboratory processing

Data on child characteristics were collected using pretested structured questionnaire from his/her parent or guardian. Then, they were instructed to provide stool sample of their child. Stool samples were collected by using sterile, wide, clean leak proof container and transported in cold box within 3 h to the Microbiology laboratory of Mekelle University.

### Culture and biochemical tests

Stool specimens were inoculated directly on to MacConkey agar (Oxoid, England) and xylose lysine deosxycholate agar (Oxoid, England) and incubated at 37 ℃ for 24 h to isolate *Shigella*. Colorless colonies from MacConkey Agar and pink colonies from xylose lysine deosxycholate agar were considered non-lactose fermenting colonies. Typical non lactose fermenting colonies were subjected to standard biochemical tests to identify *Shigella*. Biochemical tests were conducted on Kligler iron agar (KIA), Motility indole lysine medium, Simon’s citrate agar and urea. Non lactose fermenting isolates (Red slant and yellow butt) that gave negative results for gas and hydrogen sulfide on KIA, nonmotile, urea-negative, citrate negative, lysine decarboxylase and lysine deamination negative organisms were identified as *Shigella* [21].

### Statistical analysis

Data were entered, cleaned and analyzed using Statistical Package for Social Science (SPSS) 16. The Chi square test results were used to compare the prevalence of *Shigella* in different variables. P < 0.05 at 95 % confidence interval was considered as statistically significant.

### Ethical consideration

Ethical clearance was obtained from Haramaya University, Colleges of Health and Medical Sciences, Department of Medical laboratory Sciences, Ethical Review Committee. Permission to conduct this study was obtained from Mekelle zone health office and Mekelle health center. Assent was taken from children’s parents or guardians after they understood the purpose of the study.

## Results

### Characteristics of the study participants

A total of 241 diarrheic children under-5 years of age were participated in this study. One hundred thirty-four (55.6 %) of the study participants were males. The highest and lowest age group participated in the study were 0–5 (5.8 %) and 36–59 (28.2 %) months respectively.

The mean age of the study participants was 3.43 with SD ± 1.26. The duration of diarrhea shown among the study subjects was from 1 to 14 days. Majority (65.6 %) of the study participants contained blood and mucus in their stool. About 38.6 % of the study participants came to the health center within 1 day of diarrhea (Table 1).

**Table 1:** Prevalence of *Shigella* among diarrheic children under-5 years of age attending at Mekelle Health Center, North Ethiopia from March to May, 2012

Variables	Positive, no (%)	Negative, no (%)	P
Age (in months)			
0–5	0 (0)	14 (100)	0.029
6–11	2 (3.9)	49 (96.1)	
12–23	14 (22.6)	48 (77.4)	
24–35	7 (15.2)	39 (84.8)	
36–59	9 (13.2)	59 (86.8)	
Gender			
Male	17 (12.7)	117 (87.3)	0.762
Female	15 (14.0)	92 (86)	
Type of diarrhea			
Watery	8 (9.6)	75 (90.4)	NA
Bloody and mucoid	24 (15.2)	135 (84.8)	
Onset of diarrhea (days)			
1	11 (11.8)	82 (88.2)	NA
2	9 (13.8)	54 (86.2)	
3	5 (12.8)	34 (87.2)	
4–14	7 (15.9)	37 (84.1)	

### Prevalence of *Shigella*

The prevalence of *Shigella* in this study was 13.3 % (32/241). No *Shigella* was isolated from the age group of 0–5 months. The lowest (3.9 %) and highest (22.6 %) *Shigella* isolates in this study were revealed from the age group of 6–11 and 12–23 months respectively, (P < 0.05). Isolates of *Shigella* were high among females than males (P > 0.05). The highest prevalence of *Shigella* was shown among the study participants having bloody and mucoid diarrhea. The isolates of *Shigella* also high among children who came to the health center 4–14 days after their onset of diarrhea (Table 1).

## Discussion

The overall prevalence of *Shigella* in this study was 13.3 % (32/241). This was lower than the study conducted in Ethiopia: Jimma in 2001 (20.1 %) [22], and Gondar in 2009 (31.1 %) [11], but this finding is higher than studies conducted in Ethiopia in 2014: Hawassa (7 %) [14], Gondar (4.5 %) [15] and Jimma (2.3 %) [16]. Shigellosis seems to be reduced from time to time in Ethiopia as observed from 2001 by Mache [22] to studies conducted in 2014 by Getamsey et al. [14], Demissie et al. [15] and Beyene and Tasew [16].

It is also lower than studies conducted in Eritrea (47.6 %) [12], Botswana (19.5 %) [20], Nepal (79 %) [9], North Iran (76.8 %) [23] and Northeast Malaysia (30.1 %) [24].

Isolates of *Shigella* were high in the age group of 12–23 months. This might be due to the interest of children to contact with environment at a time of toddling or playing on the ground which may increase the acquisition of *Shigella* infection [25]. This is similar to the study conducted in Peruvian Amazon [26], Indonesia [27] and other developing countries studied by Platts-Mill, et al. [6]. But, it was different from the study carried out in Ethiopia: Hawassa in which the isolation rate of *Shigella* was high among the age group of 36–47 months [14]. The lowest isolation rate of *Shigella* was shown in the age group of 6–11 months with the prevalence rate of 3.9 %. This is comparable to the study conducted in northern Iran [23], but it differs from the study conducted in Ethiopia: Hawassa [14]. No *Shigella* was isolated from the age group of 0–5 months in this study. This was similar to the epidemiological study conducted in the horn of Africa [28], but it was dissimilar to the finding of the study carried out in Ethiopia: Hawassa [14] and Peruvian Amazon [26].

High prevalence of *Shigella* was isolated from the study participants whose onsets of diarrhea were between 4 and 14 days. Those diarrheic children who came late to the health center might be developed a serious feature of dehydration, abdominal cramps, even death. They might be potential source to infect other children in the community if they defecate outside the latrine.

## Limitation

This study did not perform the antimicrobial susceptibility patterns, serotypes, risk factors of *Shigella* and other diarrheal causing agents due to budget constraint.

## Conclusion

There was high prevalence of *Shigella* in this study. The most affected age group in the present study was 12–23 months. Therefore; parents or guardians, all health professionals particularly health extension workers, governmental and nongovernmental organizations should work hard on public health prevention measures. Further large scale studies also recommended in order to planning appropriate interventions by including possible risk factors associated with shigellosis in under-5 children.
